# Anethole via increase in the gene expression of PI3K/AKT/mTOR mitigates the autistic-like behaviors induced by maternal separation stress in mice

**DOI:** 10.1016/j.ibneur.2023.11.009

**Published:** 2023-11-28

**Authors:** Yasaman Yadollahi-Farsani, Vahid Reisi Vanani, Zahra Lorigooini, Anahita Farahzad, Hossein Amini-Khoei

**Affiliations:** Medical Plants Research Center, Basic Health Sciences Institute, Shahrekord University of Medical Sciences, Shahrekord, Iran

**Keywords:** Anethole, Maternal separation, Autism, PI3K/ AKT/mTOR

## Abstract

Autism spectrum disorder (ASD) is a neurodegenerative disease with increasing incidence in the world. The maternal separation (MS) stress at early life with its own neuroendocrine and neurostructural changes can provide the basis for development of ASD. Previously it has been reported neuroprotective characteristics for anethole. The PI3K/AKT/mTOR signaling pathway has pivotal role in the function of central nervous system (CNS). This study aimed to evaluate the possible effects of anethole on the autistic-like behaviors in the maternally separated (MS) mice focusing on the potential role of the PI3K/AKT/mTOR pathway. Forty male Naval Medical Research Institute (NMRI) mice were assigned to five groups (n = 8) comprising a control group (treated with normal saline) and four groups subjected to MS and treated with normal saline and or anethole at doses of 31.25, 62.5 and 125 mg/kg, respectively. All gents were administrated via intraperitoneal (i.p.) route for 14 constant days. Behavioral tests were conducted, including the three-chamber test, shuttle box and resident-intruder test. The gene expression of the PI3K, AKT and mTOR assessed in the hippocampus by qRT-PCR. Findings indicated that MS is associated with autistic-like behaviors. Anethole increased the sociability and social preference indexes in the three-chamber test, increased duration of secondary latency in the shuttle box test and decreased aggressive behaviors in the resident-intruder test. Also, anethole increased the gene expression of PI3K, AKT and mTOR in the hippocampus of MS mice. We concluded that anethole through increase in the gene expression of PI3K/ AKT/mTOR mitigated autistic-like behaviors induced by MS in mice.

## Introduction

Autism spectrum disease (ASD) is a neurodevelopmental disorder associated with impedes social interactions and communications as well as repetitive and restricted behaviors (Porter et al., 2022, Johansson and Sandin, 2023). Studies have demonstrated that various factors such as epigenetic, metabolic and environmental conditions are ASD pathogenic factors (Rødgaard et al., 2021). Maternal separation (MS) stress is a commonly used animal model of early-life stress (ELS) (Own and Patel, 2013). According to the previous studies, it has been determined that exposure of neonates to MS stress, disrupt brain development as well as lead to neurostructural, neurochemical and neurohormonal alterations, consequently increases the susceptibility to neuropsychiatric disorders (Lorigooini et al., 2021, Arabi et al., 2021, Anjomshoa et al., 2020). Hippocampus has pivotal role in the pathophysiology of ASD (Liu et al., 2022, Jiang et al., 2023). Growing evidences demonstrated that MS stress is associated with autistic-like behaviors in rodents (Makris et al., 2023, Farzan et al., 2023, Moghaddam et al., 2023). However, the exact mechanisms underlying the association between MS stress and autistic- like behaviors did not fully understand yet.

Anethole (1-methoxy-4-propenylbenzen) is a natural component found in plants like anise, fennel, and dill (Yu et al., 2022). Previous studies have been reported various pharmacological effects for anethole including antioxidant, anti-inflammatory, anti-nociceptive and antiepileptic properties (da Guedes et al., 2022, Kang et al., 2013, Ritter et al., 2013, Ghasemi-Dehnoo et al., 2022, Pandit et al., 2022). Previous studies have been demonstrated that anethole possessed neuroprotective effects in various neurological disorders (Vastegani et al., 2023, Chang et al., 2022, Salimian et al., 2022, Raman et al., 2020). It has been determined that anethole attenuated anxiety- and depressive-like behaviors as well as improved memory in socially isolated rats (Raman et al., 2020). Considering that there is no definitive and approved treatment for ASD (Qu et al., 2022) and based on abovementioned neuroprotective effects reported for anethole, this study designed to evaluate potential effect of anethole on autistic-like behaviors induced by MS in male mice.

The PI3K/Akt/mTOR signaling pathway is divided into two branches. The first is phosphatidylinositol 3 (PI3K) and serine-threonine protein B (PKB; or AKT) and the second branch is mammalian target of rapamycin (mTOR) (Bilanges et al., 2019). The PI3K/Akt/mTOR signaling pathway has fundamental role in neurogenesis, synaptogenesis, and neuronal plasticity (LiCausi and Hartman, 2018, Borrie et al., 2017). Several studies have reported that PI3K has role in synaptic plasticity, learning and memory as well as involved in the pathophysiology of psychiatric disorders (Ludka et al., 2016, Pazini et al., 2016). It has been determined that dysregulation and mutation in the PI3K/AKT/mTOR signaling pathway led to progression of autistic-like behaviors (Chen et al., 2014). It has been shown that disruption in the mTOR signaling is association with the development of ASD (Thomas et al., 2023). Down-regulation of the Akt/mTOR pathway has been reported in rodents with autistic-like behaviors (Nicolini et al., 2015, Russo, 2015). Some evidence has shown contrary to this issue which inhibition of the PI3K-AKT/mTOR signaling pathway by chrysophanol attenuated autistic behaviors in rats (Sharma et al., 2022). However, the molecular mechanisms underlying in ASD especially the exact role of PI3K-AKT/mTOR signaling pathway remain to be clarify.

In this study we aimed to evaluate firstly possible changes of the PI3K-AKT/mTOR signaling pathway in the hippocampus following MS-induced autistic-like behaviors and in second evaluate possible changes of this pathway following administration of anethole in MS mice.

## Material and methods

### Animals and maternal separation paradigm

Fifteen pregnant Naval Medical Research Institute (NMRI) mice (first-day pregnancies) were bought from the Pasteur Institute in Iran. The standard maintenance conditions included a temperature of 21 ± 2 ° C, a 12-hour dark/light cycle, and free access to water and food were provided. The mice gave birth (an average of 8 pups per mouse with an average weight of about 3 g at birth and about 50% male and 50% female pups). The first day of life was considered postnatal day zero (PND 0). The pups were subjected to maternal separation (MS) paradigm on PND2. Offsprings were separated from their mothers three hours daily until PND14 and then reverted to their mother cages. From PND14–21, the pups were persisted in the mother's cage without manipulation or separation. Finally, on PND21, male mice (from different mothers) rendomly were divided into four groups (n = 8). Additionally, eight NMRI male mice that were not exposed to the MS model were selected as the control group. Treatments with saline or anethole were began between PND 45 up to 60 for 14 constant days and then mice subjected to behavioral tests. The sample size was considered by power calculations using G power software (ver.3.1.7, Franz Faul, Universitat Kiel, Germany). We fixed α error at 0.05 and the power of the study at 0.8 and the prerequisite sample size in each group was calculated 8 for behavioral tests and 4 for molecular assessments (Haj-Mirzaian et al., 2017).

### Study design

**Fig. 1:**
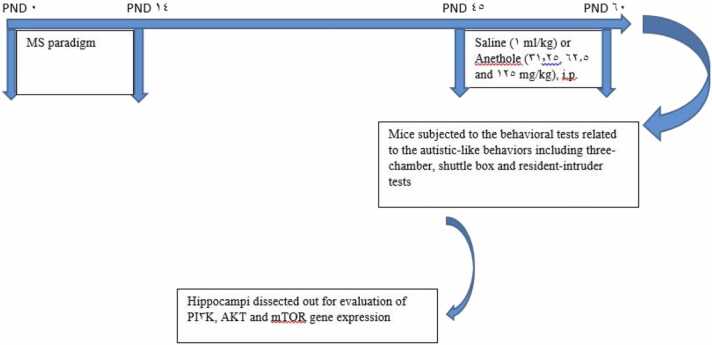
Schematic of the study design.

Forty male NMRI mice were divided in 5 experimental groups (n = 8) as follows: Groups 1–4 were maternally separated mice received normal saline (1 ml/kg) or anethole at doses of 31.25, 62.5 and 125 mg/kg, respectively. Group 5 was control group (without MS stress) received normal saline (1 ml/kg). All agents were administrated intraperitoneally (i.p.) for 14 constant days between PND 45–60. The dose and time of agent administrations were chosen based on previous studies and our pilot study (Ghasemi-Dehnoo et al., 2022, Salimian et al., 2022, Wisniewski-Rebecca et al., 2015). Then mice were subjected to behavioral tests related to autistic-like behaviors including three-chamber sociability, shuttle box and resident-intruder tests. Then mice were euthanized (deep anesthesia by pentobarbital (60 mg/kg, i.p.) (Amiri et al., 2017) and considering role of hippocampus in the modulation of autistic symptoms (Walsh et al., 2022, Pignataro et al., 2023), the hippocampi were dissected on the ice-cold surface and directly placed into liquid nitrogen and then stored at −80 °C for molecular evaluations. The schematic of the study design is shown in Fig. 1.

## Behavioral tests

### Shuttle box test

This test was performed in order to investigate the passive avoidance memory. Passive avoidance memory was assessed as previously described (Tavassoli et al., 2023). Briefly, the mice were placed in an apparatus with a bright and a dark chamber linked with a door. Adaption of mice was done in the first two days; mice were allowed to explore the apparatus for 5 min individually. On the third day, the mice were left in the bright chamber, and after 2 min, the door was opened; when mice entered into the dark chamber, the door was closed, and an electrical shock (1 mA/second) was imposed. The latency to enter the dark chamber was considered the initial latency (T1). The same procedure was done on the fourth day without electrical shock, and the second latency was documented (T2).

### Resident-intruder test

Offensive aggression was assessed by resident- intruder test as previously described (Koolhaas et al., 2013). Briefly, resident male mice were placed with a companion female in a resident 30 × 20×13 cm plastic cage for a week. The companion was removed, and one hour later, an unfamiliar smaller male mouse was introduced to the resident cage while food and water bottle were removed from the cage. The behavior of the resident subject was recorded for 10 min. The duration of offensive behaviors documented manually by an experimenter who was blind to the groups. Time which the resident mouse passes attacking the intruder as well as time of rearing, non-social exploration and self-grooming behaviors were documented. The following behaviors are recorded as offensive behaviors (attacks): bites, lateral threat, upright posture by the resident, clinch, keep down, tail rattles and chase.

### Three-chamber sociability test

To assess social behaviors, we utilized the three-chamber test. Apparatus was a plexiglass box 102 cm (length) × 47 cm (width) × 45 cm (height) partitioned into three chambers, including a middle chamber and two side chambers. The size of two side chambers that the stimuli are placed was 33 cm (length) × 47 cm (width). In order to house the social or non-social stimulus to perform the sociability phase a cylindrical wire cup (made of sturdy steel) in measure 10.2 cm (diameter) × 10.8 cm (height), with 1 cm gaps between bars to provide adequate area for mice interaction and sniffing was placed in each side chamber. Throughout the habituation, sociability, and social preference phases, the mice were given the freedom to explore the box. In the habituation phase, mice were placed in the middle chamber for 5 min to acclimatize themselves to the surroundings. To perform the sociability phase, one wire cage had a same-sex and same-age mouse that had no prior interaction with the subject mice placed inside it (novel mice 1 or social stimulus 1), and the time spent to explore each chamber was measured for 10 min. The other wire cage remained empty. The amount of time spent directly interacting with the social stimulus and the empty chamber (non-social stimulus) was recorded. The sociability index (SI) was calculated as (social stimulus 1 - non-social stimulus)/ (social stimulus 1 + non-social stimulus). In the social preference stage (SPI), a novel mouse (new mice 2 or social stimulus 2) with characteristics similar to those of the novel mice 1 was placed in another empty wire cage, and the amount of time spent in each chamber was recorded for 10 min. The SPI was determined using the following formula: (social stimulus 2 - social stimulus 1)/ (social stimulus 2 + social stimulus 1). All recording performed by an experimenter blinded to the study. The following behaviors calculated as direct interactions with the stimulus mouse or non-social object; sniffing the base of the cup containing the stimulus, interacting with parts of the stimulus (such as tail) that are protruding from the wire cup and aggressively attending to (sniffing/facing) the stimulus while climbing the cup (Rein et al., 2020, Huang et al., 2019).

#### Quantitative real-time polymerase chain reaction (qRT-PCR)

Hippocampus was removed and immediately frozen in liquid nitrogen and stored at −80 °C until assessment. Total RNA was extracted from hippocampi by Trizol reagent (Invitrogen, Cergy Pontoise, France). Alterations in the mRNA levels of genes were determined using qRT-PCR as described previously (Nouri et al., 2020). Briefly, the reverse transcription of 1 μg of RNA from each sample was done using the PrimeScript RT reagent kit (Takara Bio Inc., Otsu, Japan). qRT-PCR was completed on a light cycler device (Roche Diagnostics, Mannheim, Germany) using SYBR Premix Ex Taq technology (Takara Bio). Sequences of primers are shown in Table 1. B2m was considered a normalizer, and fold changes in expression of each target mRNA relative to B2m were calculated based on the 2-ΔΔCt relative expression formula.

**Table 1:** Sequences of qRT-PCR specific primers.

Primer	Forward sequence	Reverse sequence
PI3K	GCAACTCCTGGACTGCAACT	CAGCGCACTGTCATGGTATG
AKT	TAGCCATTGTGAAGGAGGGC	CCTGAGGCCGTTCCTTGTAG
mTOR	GCTCCAGCACTATGTCACCA	CGTCTGAGCTGGAAACCAGT
B2m	GGAAGTTGGGCTTCCCATTCT	CGTGATCTTTCTCGTGCTTGTC

### Statistical analysis

GraphPad Prism software (Version 8) was used for data analysis. Kolmogorov–Smirnov test was applied to evaluate the normal distribution of data. Using Brown-Forsythe test, homogeneity of variances has been checked. Statistical analysis of the data was conducted through a one-way analysis of variance (ANOVA), followed by Tukey’s multiple comparison test. Results were deemed statistically significant at p < 0.05.

## Results

### Anethole improves passive avoidance memory in the shuttle box test

**Fig. 2:**
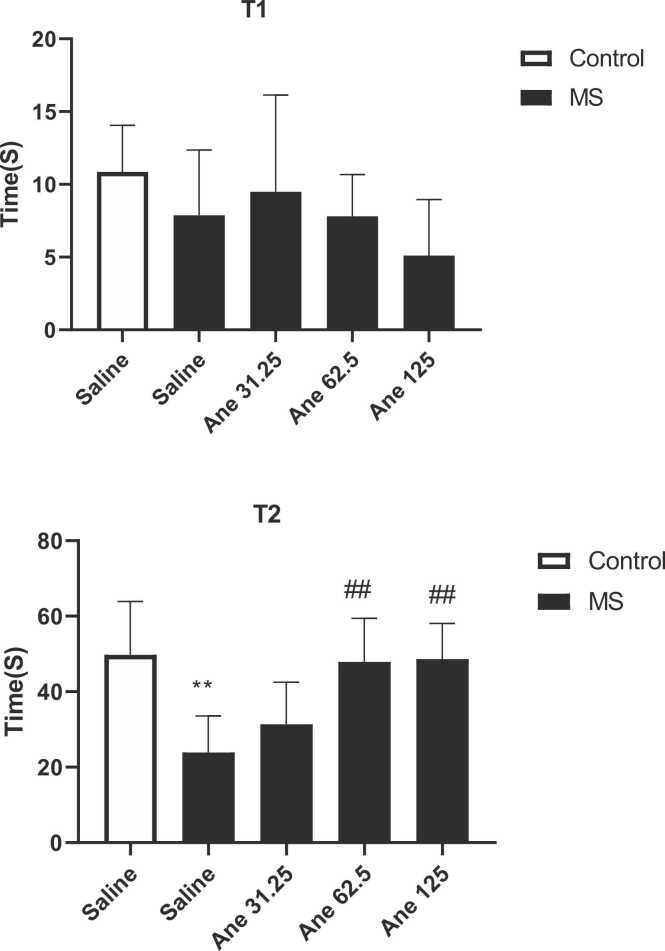
Comparison of the initial (T1) and secondary (T2) delays in entering in to the dark box in the shuttle box test**.** Data are presented as Mean ± SEM., from 8 mice and were analyzed with one-way ANOVA followed by tukey’s post test. **P < 0.01 compared with the saline-treated control group and ^##^P < 0.01 compared with the saline-treated MS group. Ane: anethole, MS: maternal separation.

According to the one-way ANOVA, the initial delay duration (T1) did not show any significant difference among the experimental groups (F (Own and Patel, 2013, Wisniewski-Rebecca et al., 2015) = 1/449, P = 0/2536, Fig. 2). The results showed that there was no notable difference in the initial phase (T1) of the shuttle box test between experimental groups. One-way ANOVA analysis determined that there are significant difference among experimental groups in case of secondary delay duration (T2) in the shuttle box test (F (Own and Patel, 2013, Haj-Mirzaian et al., 2017) = 6/948, P = 0/0009). Post test analysis on secondary delay duration (T2) showed that in the saline-treated MS group, the T2 was significantly reduced in compared to the control group (P < 0.01). In addition, the T2 in the MS mice received anethole at doses of 62.5 and 125 mg/kg significantly increased in compared to the saline-treated MS mice (P < 0.01) (Fig. 2).

### Anethole reduces aggressive behaviors in the resident-intruder test

**Fig. 3:**
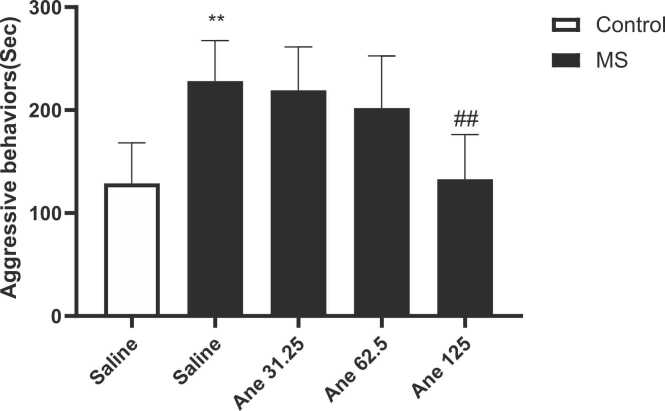
Comparing the aggressive behaviors in the resident-intruder test. Data are presented as Mean ± SEM., from 8 mice and were analyzed with one-way ANOVA followed by tukey’s post test. **P < 0.01 Compared to the saline-treated control group and ##P < 0.01 compared to the saline-treated MS group. Ane: anethole, MS: maternal separation.

One-way ANOVA analysis showed significant difference in the aggressive behaviors among studied groups (F (Own and Patel, 2013, Sharma et al., 2022) = 7/659,P = 0/0005, Fig. 3). We observed that the aggressive behavior significantly increased in the saline-treated MS mice in compared to the control group (P < 0.01). Moreover, aggressive behaviors following administration of anethole at dose of 125 mg/kg significantly decreased in compared to the saline-treated MS mice (P < 0.01).

### Anethole improves social behaviors in the three-chamber sociability test

**Fig. 4:**
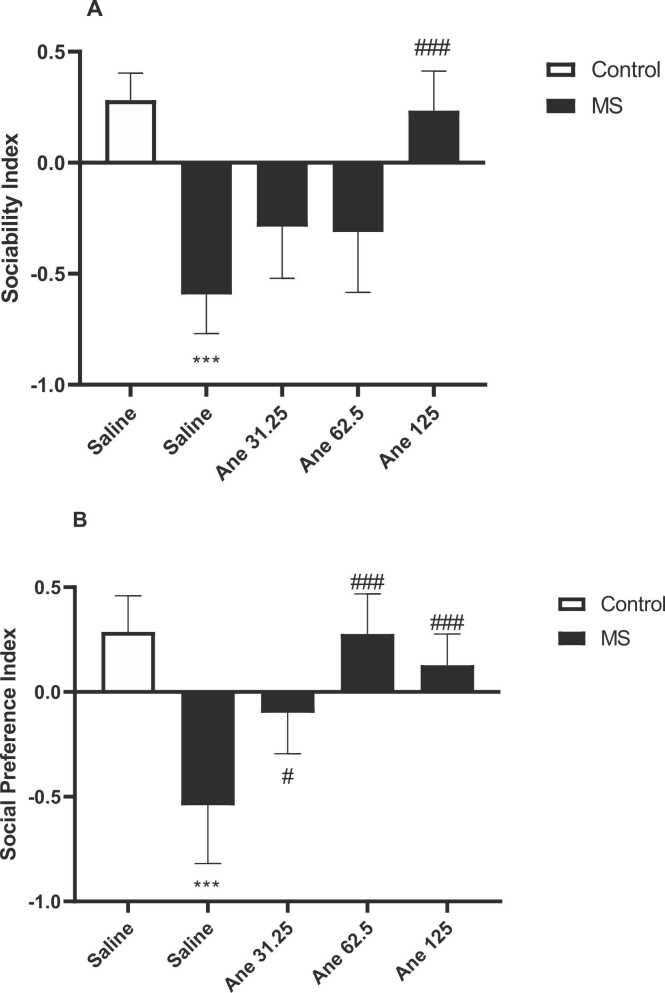
Social behaviors in the three-chamber sociability test. A: sociability index (SI) and B: social preference index (SPI)**.** Data are presented as Mean ± SEM., from 8 mice and were analyzed with one-way ANOVA followed by tukey’s post test. * **P < 0.001 Compared to the saline-treated control group, #P < 0.05 and ###P < 0.001 compared to the saline-treated MS group. Ane: anethole, MS: maternal separation.

Based on the one-way ANOVA, there are significant differences in cases of SI (F (Own and Patel, 2013, Russo, 2015) = 21/61, P < 0/0001, Fig. 4A) and SPI (F (Own and Patel, 2013, Russo, 2015) = 15/65, P < 0/0001, Fig. 4B) among experimental groups in the three chamber test. SI following MS significantly decreased in compared to the control group (P < 0.001). In addition, we found that administration of anethole at dose of 125 mg/kg to the MS mice significantly increased the SI in compared to the saline-treated MS mice (P < 0.001).

Our results showed that SPI following MS significantly decreased in compared to the control group (P < 0.001). Furthermore, administration of anethole at doses of 31, 25 (P < 0.05), 62.5 (P < 0.001) and 125 mg/kg (P < 0.001) to the MS mice significantly increased the SPI in compared to the saline-treated MS mice.

### Anethole increases the gene expression of PI3K, AKT and mTOR in the hippocampus

**Fig. 5:**
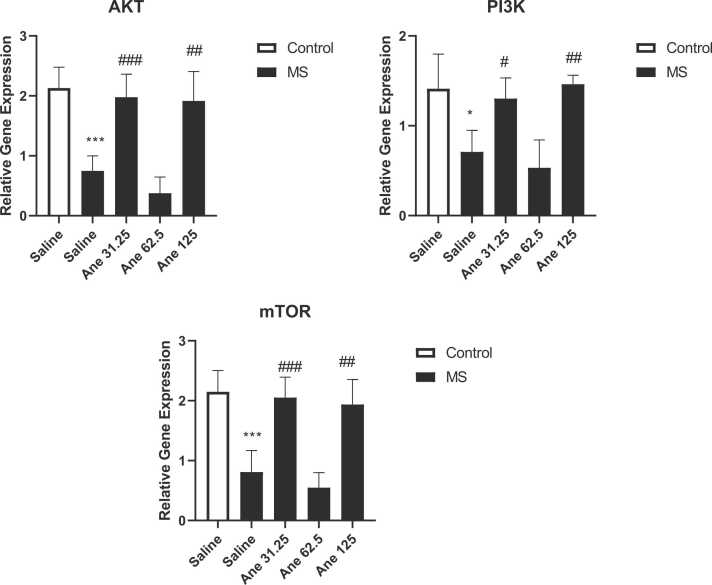
the gene expression of PI3K, AKT and mTOR in the hippocampus**.** Data are presented as Mean ± SEM., from 4 samples and were analyzed with one-way ANOVA followed by tukey’s post test. *P < 0.05 and * **P < 0.001 compared to the saline-treated control group, #P < 0.05, ##P < 0.01 and ###P < 0.001 compared to the saline-treated MS group. Ane: anethole, MS: maternal separation.

Based on the one-way analysis of variance, there are significant differences in case of gene expression of PI3K (F (Own and Patel, 2013, Kang et al., 2013) = 11/62, P < 0/0001), AKT (F (Own and Patel, 2013, Kang et al., 2013) = 26/01, P < 0/0001) and mTOR (F (Own and Patel, 2013, Kang et al., 2013) = 24/59,P < 0/0001) in the hippocampus among experimental groups (Fig. 5). Results showed that the gene expression of PI3K (P < 0.05), AKT (P < 0.001) and mTOR (p < 0.05) in the hippocampus significantly decreased in the MS mice in compared to the control mice. Our results showed that administration of anethole at doses of 31.25 (P < 0.05) and 125 mg/kg (P < 0.01) significantly increased the gene expression of PI3K in the hippocampus of MS mice in compared to the saline-treated MS mice. We observed that administration of anethole at doses of 31.25 (P < 0.001) and 125 mg/kg (P < 0.01) significantly increased the gene expression of AKT in the hippocampus of MS mice in compared to the saline-treated MS mice. Finding determined that administration of anethole at doses of 31.25 (P < 0.001) and 125 mg/kg (P < 0.01) significantly increased the gene expression of mTOR in the hippocampus of MS mice in compared to the saline-treated MS mice. We did not observe significant difference in case of gene expressions at dose of 62,5 mg/kg in compared to the control group.

## Discussion

Our results showed that MS stress provoked autistic-like behaviors in male mice. We observed that following MS stress SI and SPI decreased in three chamber sociability test as well as passive avoidance memory impaired in the shuttle box test and aggressive behaviors increased in the resident-intruder test. In aspect of molecular evaluations in the hippocampus, we observed that MS is associated with decreased in the gene expression of PI3K, AKT and mTOR. Finding determined that anethole reversed abovementioned negative effects of MS on behaviors as well as increased the gene expression of PI3K, AKT and mTOR in the hippocampus.

ASD is a neurodevelopmental disorder characterized by a deficiency in social behaviors, verbal and non-verbal communication and relationships, aggressive behaviors and memory impairment (Lorigooini et al., 2021, Goswami et al., 2021, Liu et al., 2023, Loftus et al., 2023). These collections of neurodevelopmental behaviors mediated through neural mechanisms which are mainly unknown completely up to now. It has been suggested that genetic background and alteration in gene expressions mediate these neural mechanisms (Lee et al., 2012, Arakawa, 2021). It has been reported that ASD is associated with structural abnormalities in the hippocampus (Xu et al., 2020, Doyle-Thomas et al., 2014). In this regard Arutiunian et al., reported that reduced in grey matter volume of hippocampus is linked with the severity of ASD symptoms in children (Arutiunian et al., 2023). Furthermore various studies have demonstrated role of neural and signaling alterations of the hippocampus in the pathophysiology of ASD (Dutra et al., 2023, Zohny et al., 2023, Guo et al., 2023).

Previously it has been well-determined that experiencing early life stress impose negative effects on brain development and function which subsequently interrupt the behavior, thought emotions and memory (Andersen, 2022). As an accepted model, MS lead to structural, developmental, neuroendocrine and neurochemical alterations in different areas of the brain including hippocampus. These alterations result in mood/behavioral disorders as well as neurodegenerative diseases in the adulthood (Sierra-Fonseca et al., 2021, Tanaka et al., 2021). Previous studies have determined that early life stress like MS is associated with autistic-like behaviors. In this regards it has been reported that MS stress is associated with lower SI and SPI in the three chamber sociability test as well as lower step-through latency (T2) in the shuttle box test (Farzan et al., 2023, Ren et al., 2022, Sayed Javad Javaheri et al., 2019). In line with abovementioned studies, we observed that MS stress decreased secondary delay time in the shuttle box test, increased aggressive behaviors in the resident-intruder test and decreased SI and SPI in the thre-chamber sociability test.

The PI3K/Akt/mTOR signaling pathway has essential role in the regulation of neural function (Iranpanah et al., 2023). In the recent years, several pieces of evidence have determined that dysregulation of this signaling pathway in associated with ASD (Chen et al., 2014). Down-regulation of PI3K-AKT/mTOR signaling pathway is linked with autistic symptoms (Thomas et al., 2023, Nicolini et al., 2015, Russo, 2015). Sheikh et al., clarified that down-regulation of Akt decrease the concentration of brain-derived neurotrophic factor (BDNF) in the autistic brain which could be consider as one of the underlying mechanisms in the pathophysiology of autism (Sheikh et al., 2010). Seo et al. have demonstrated that increase in the expression of PI3K and p-Akt in the hippocampus attenuated autistic-like behaviors in the valproic acid-induced autism in rats (Seo et al., 2013). In contrary some evidences showed that inhibition of the PI3K-AKT/mTOR signaling pathway may attenuate autistic behaviors (Sharma et al., 2022, Sharma and Mehan, 2021). Kwon *et al.*, showed that abnormal activation of the PI3K/AKT pathway in the hippocampus is associated with behavioral abnormalities related to the ASD (Kwon et al., 2006). However, the exact role of PI3K-AKT/mTOR signaling pathway in the autism remain to be clarify. Our results showed that autistic-like behaviors following MS stress is accompanied with decrease in the gene expression of PI3K, AKT and mTOR in the hippocampus. Indeed, more studies warranted in the future to exactly clarify role of this signaling pathway in the pathophysiology of ASD. Furthermore, future studies warranted to evaluate change in the PI3K, AKT and mTOR in the protein level. One limitation of our study was to we evaluate change in this pathway in the gene level. Further studies warranted in the future to evaluate changes in the PI3K-AKT/mTOR signaling pathway following MS at the protein level.

Anethole (1-methoxy-4-propenylbenzene), a major component of fennel oil, has been exerted various pharmacological properties including anti-oxidative stress, neuroprotective, anticonvulsant, anti-inflammatory, anxiolytic and anti-depressant effects (Ghasemi-Dehnoo et al., 2022, Vastegani et al., 2023, Salimian et al., 2022, Alvarado-García et al., 2022, Wang et al., 2018, Bhatti et al., 2018). Raman *et al.*, reported that anethole improved spatial memory in rats subjected to social isolation stress (Raman et al., 2020). Our results showed that administration of anethole significantly reversed the negative effects of MS on the behaviors. We showed that anethole increased the secondary delay time in the shuttle box test, decreased the aggressive behaviors in the resident-intruder test and increased the SPI and SI in the three-chamber sociability test. Furthermore, in case of molecular assessments, we found that following treatment with anethole, the gene expression of PI3K, AKT and mTOR significantly increased in the hippocampus of the MS mice. Overall, finding of the present study suggesting that anethole via increase in the gene expression of PI3K, AKT and mTOR in the hippocampus mitigated the autistic-like behaviors in MS mice. However, more studies warranted in the future to find the exact mechanisms involved in the anti-autistic like effects of anethole.

## Conclusion

We concluded that anethole through increase in the genes expression of PI3K, AKT and mTOR in the hippocampus attenuated the autistic-like behaviors induced by MS stress in male mice.

## Ethical approval

All steps of experimentation were performed in accordance with the regulations of the Shahrekord University of Medical Sciences Guide for the Care and Use of Laboratory Animals (Ethics code: IR.SKUMS.REC.1399.238). Full determinations were made to reduce the use of animals and to advance their comfort.

## Funding

This study was supported by a research grant (3532) from 10.13039/501100005756Shahrekord University of Medical Sciences, Shahrekord, Iran.

## CRediT authorship contribution statement

**Yasaman Yadollahi-Farsani:** Investigation, Formal analysis, Visualization, Writing – original draft. **Vahid Reisi Vanani:** Methodology, Investigation. **Zahra Lorigooini**: Methodology, Investigation. **Anahita Farahzad**: Methodology, Investigation. **Hossein Amini-Khoei**: Conceptualization, Supervision, Resources, Writing – review & editing, Project administration, Funding acquisition.

## Declaration of Competing Interest

The authors have no conflicts of interest to declare regarding the study described in this article and preparation of the article.

## Data Availability

The data used to support the findings of this study are available from the corresponding author upon request.
